# A Focused Review of Language Use Preceding Death by Execution

**DOI:** 10.3389/fpsyg.2018.00683

**Published:** 2018-05-15

**Authors:** Sarah Hirschmüller, Boris Egloff

**Affiliations:** Department of Psychology, Johannes Gutenberg University Mainz, Mainz, Germany

**Keywords:** language use, text analysis, execution, death, emotion regulation

## Abstract

Executions are one form of death that can be assumed to be maximally anxiety provoking. Words spoken by death row inmates moments before their execution can provide valuable insights into people's end-of-life communication needs and themes, conveying what individuals choose to express to others in the face of imminent death. In this focused review, we describe findings from quantitative and qualitative text analysis studies that have analyzed affective experiences and meaning-making attempts in transcriptions of actual statements made by Texas death row inmates. Overall, the most prevalent content themes identified in these final acts of verbal communication in the reviewed studies consisted of a strong predominance of emotional positivity, messages to relevant social others, and spiritual references. We subsequently view the reviewed findings in the light of additional research in which people's conceptions of death and dying were explored and language studies in which people's communication before other forms of death was analyzed. Finally, we describe open questions and directions for future analyses of death row inmates' final statements, and we outline practical implications.

## Introduction

Death and dying are an inevitable part of the human experience, with people's imaginations of death having inspired countless literary texts, paintings, and music, as well as substantial psychological research (e.g., Becker, 1973; Kastenbaum, 2000). People's general ideas about death appear to be extremely fear-laden and anxiety-provoking—when envisioning death and dying, people dread, for example, the possibility of pain and suffering, the loss of loved ones, and feelings of meaninglessness (Neimeyer, 2015). What feelings and personal concerns are expressed by individuals who actually have only minutes left to live? The last words of dying people, having fascinated the general public (Marvin, 1901) and psychological researchers (e.g., Kübler-Ross, 1969) for a long time, can provide valuable insights into people's affective experiences and meaning-making when encountering actual death. Last words in the face of actual death have been recorded as early as 1388 in England (Howell, 1809) as part of the execution process. Even today, the last words of people facing execution are collected and made public by the US State of Texas (Texas Department of Criminal Justice, 2017a). In this focused review, we provide a comprehensive overview of studies that have examined expressions of affect and meaning-making in executed death row inmates' last words. Next, we discuss the implications of the reviewed findings in the light of other research on death and dying. The article concludes with a description of open questions and suggestions for how such types of utterances can be examined in the future and a brief overview of practical implications.

## Analyzing the phenomenon: language use in last words before execution

With the **death penalty** not yet abolished in many parts of the world today, executions—characterized by a complete absence of controllability and subjection to powerful others who have the authority to end one's life (Hood and Hoyle, 2015)—are one form of death that can be assumed to be maximally anxiety-provoking. The US is the only Western country that still enforces the death penalty (for a detailed history of the death penalty in the US, see Banner, 2003), and moral, legal, and ethical issues of these executions have been the focus of much political debate (Bedeau and Cassell, 2004). Currently, the death penalty is legal in 31 states, but since 1976, more than one third of all 1,465 executions in the US have occurred in Texas (Death Penalty Information Center, 2017). As part of the execution process, death row inmates in Texas are allowed a last statement, and transcripts are made public after death on the Texas Department of Criminal Justice (2017a) website. In the presence of the warden and chaplain, the last words may be spoken into a microphone after the inmate has been strapped to the gurney in the execution chamber. When the inmate has finished the statement, the warden signals for the lethal injection to begin (Texas Department of Criminal Justice, 2012). Seated in separate rooms, media representatives and friends and relatives of the inmates or victims are allowed to witness executions (Texas Department of Criminal Justice, 2017b). What emotions and personal concerns do death row inmates verbally express in the face of imminent actual death? To what extent do the last words of executed prisoners reflect the dread and anxiety we intuitively ascribe to death and dying?

KEY CONCEPT 1 **Death penalty**

A government sanctioned practice, also known as capital punishment, which has a long and controversial history in the US, whereby a person sentenced to death is executed by the state as a punishment for a capital crime.

### Affective experiences and meaning-making in executed death row inmates' last words

**Figure 1:**
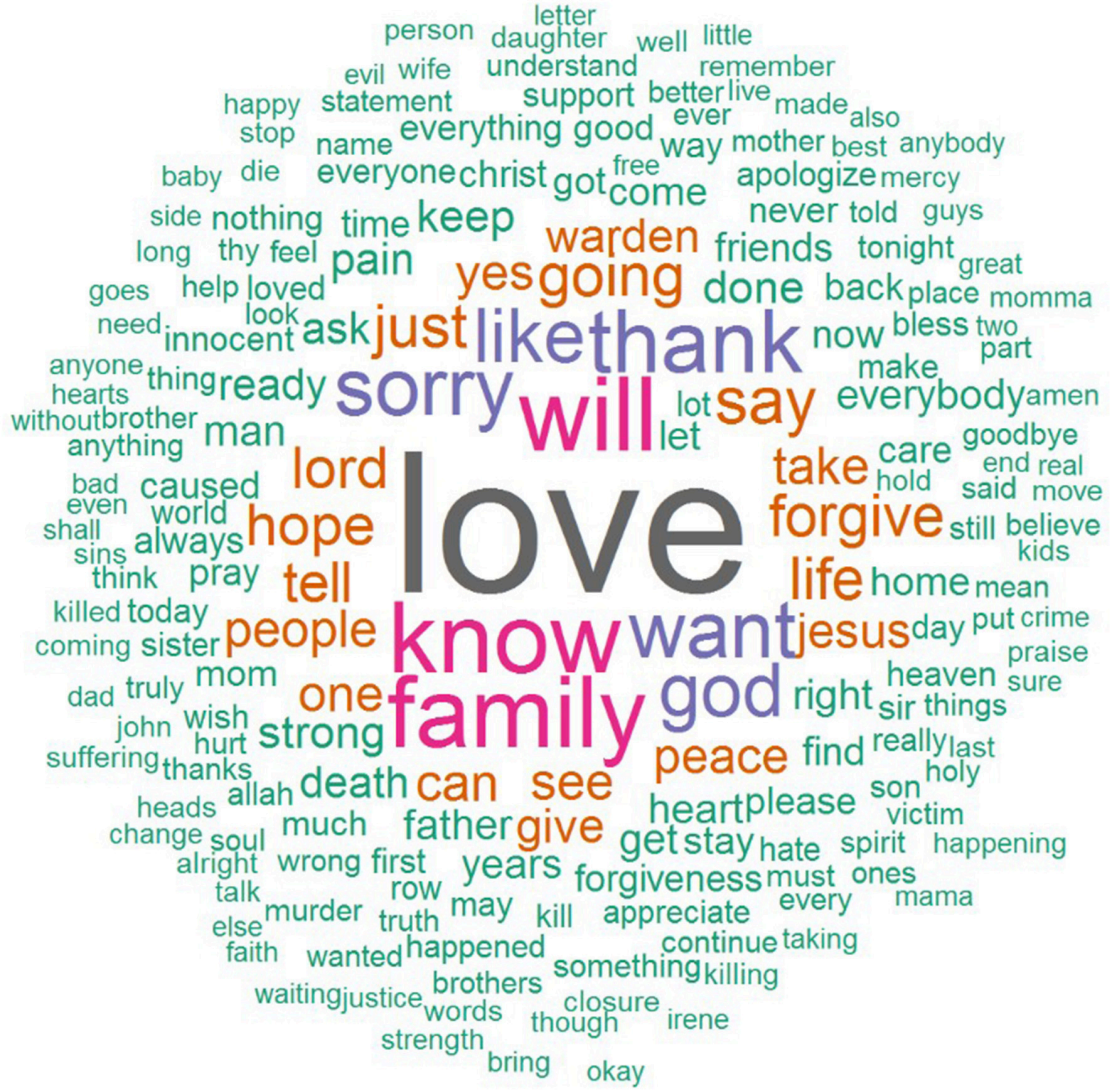
Words most commonly used by death row inmates executed between 1982 and 2015 (June, 30) in Texas.

Contributing to the psychological understanding of affective regulation and meaning-making mechanisms in the face of actual death, a number of text analysis studies have explored word choice and content themes in executed prisoners' last words. In a recently published study (Hirschmüller and Egloff, 2016), we used **quantitative text analysis** (Mehl, 2006) to examine expressions of affect and content themes in executed Texas death row inmates' last words with the text analysis program Linguistic Inquiry and Word Count (LIWC; Pennebaker et al., 2007a; most recent version by Pennebaker et al., 2015). LIWC compares words against an internal default dictionary, and besides capturing the total number of words and general descriptors (e.g., percentage of words identified by the internal dictionary), it counts the percentages of words falling into linguistic categories (e.g., personal pronouns, verbs), psychological categories (e.g., words reflecting affective, social, or cognitive processes), and specific content categories (e.g., religion, death) with adequate psychometric properties (e.g., Kahn et al., 2007; Pennebaker et al., 2007b; Bantum and Owen, 2009). Of the 527 executions between December 1982 and June 2015 regarded in our study, 119 of the inmates had declined to make a last statement or had no transcribed last statement (Texas Department of Criminal Justice, 2017a). Of the 407 executed prisoners (404 male, 3 female) with spoken last words, 178 were described as White or Caucasian, 150 as Black, 77 as Hispanic, and two as having another ethnicity. Their mean age at execution was 39.01 (*SD* = 8.2) years, and they had been on death row for an average of 10.96 (*SD* = 4.4) years. Death row inmates' statements contained a total of 42,328 words and an average of 104 words per inmate (ranging from 1 to 1,268 words) of which, on average, 92% were categorized by the internal LIWC dictionary. It is interesting that the final statements included, on average, a higher percentage of positive emotion words (9.64%) than negative emotions words (2.65%), with over 80% of death row inmates speaking more positive than negative emotion words. The proportion of positive emotion words was significantly related to death row inmates' greater use of first-person singular self-references, words reflecting social orientation including words referring to friends, and present-tense verbs, as well as to less use of words indicating cognitive processes, past-tense verbs, and death-related words. Figure 1 shows the words most commonly used by the 407 executed prisoners included in our study (Hirschmüller and Egloff, 2016) in the form of a word cloud in which the sizes of the words reflect their frequency. Please note that the words most commonly used in English (i.e., stopwords) were not included in the word cloud, which was created using the R package “wordcloud” (Fellows, 2015).

KEY CONCEPT 2 **Quantitative text analysis**

A class of textual analysis methods that are applied to assess quantitative information about the presence, intensity, or frequency of stylistic or thematic characteristics in textual material and that can be subjected to statistical analysis.

A number of **qualitative text analysis** (Kuckartz, 2014) studies have relied on the same data source of final statements from the Texas Department of Criminal Justice (2017a) website from varying time periods and used ratings by humans to identify expressions of affect and prevalent content themes in these statements (e.g., Heflick, 2005; Foley and Kelly, 2007; Schuck and Ward, 2008; Vollum and Longmire, 2009; Kelly and Foley, 2013). Content themes in 237 last statements spoken by Texas death row inmates executed between January 1997 and April 2005 identified by Heflick (2005) without quantifying the frequency were love/appreciation, forgiveness, claims of innocence, belief in an afterlife, activism (e.g., advising people in the inmate's life or appeals to end the death penalty), and silence as some inmates chose not to give a last statement. Foley and Kelly (2007) also categorized the themes in 100 last statements made by Texas death row inmates executed between April 2002 and November 2006 and found nine themes, the most common being references to love (70%), spirituality (56%), apologizing to the victim's family (37%), and regret for the offense (36%). In a subsequent thematic analysis of 79 final statements spoken between December 2006 and July 2011 (Kelly and Foley, 2013), love (82%), spirituality (52%), regret for the offense (39%), and apologizing to the victim's family (37%) were also rated as the most prevalent themes. Similarly, Vollum and Longmire (2009) explored major themes and subsets of more specific themes in final statements from 292 executions between December 1982 and March 2004 and found that the most common of the 10 major themes that were identified included well-wishes and expressions of love (59%), religion (48%), contrition (33%), and gratitude (30%; see also Vollum, 2008).

KEY CONCEPT 3 **Qualitative text analysis**

A class of textual analysis approaches that use a wide spectrum of non-standardized methods to extract qualitative information about the presence, relations, or structural patterns of stylistic or thematic characteristics from textual material that can be variously interpreted.

Schuck and Ward (2008) identified 13 prominent themes of which the most common ones were expressions of love/appreciation (63%), addressing others (i.e., family members, friends, or the victim's family, 55%), religion (46%), seeking forgiveness (39%), and expressions of self-comfort (39%) in the final statements of 283 death row inmates executed between December 1982 and November 2006. Subsequently, they applied a discourse-analytic approach to analyze the sequential structuring and patterns in these statements. According to the authors, the final statements most commonly began with a self-reference, followed by messages directly toward family members and friends or the victim's family, expressions of internal feelings, and references to the situation, either an acceptance of it (e.g., by taking responsibility) or a rejection of it (e.g., by declaring their innocence or making political statements). Subsequently, inmates' statements most often indicated how they handled the situation, including outward-oriented (e.g., comforting others, seeking forgiveness, wishes for others) and/or inward-oriented (e.g., expressions of religion or self-comfort) expressions in the case of acceptance, and only rarely expressions of denial or accusations, commonly followed by a last sentence in the form of simple closure.

In addition, several qualitative studies pursuing a rather specific thematic focus have examined apology and remorse in final statements spoken by death row inmates before execution (e.g., Eaton and Theuer, 2009; Rice et al., 2009, see also Cooney and Phillips, 2013; Eaton, 2014). Examining the frequency and content of apologies, Eaton and Theuer (2009), for example, reported that 33% of the 402 prisoners executed between December 1982 and August 2007 in Texas offered an apology for their crime, most of which were directed toward the victim's family. In addition, inmates' apologies were often accompanied by expressions of sincerity and remorse in their last statements, with inmates admitting guilt for the crime (23%), asking for forgiveness (21%), or showing empathy for the victim's family (26%). Rice et al. (2009), regarding executions that were carried out from December 1982 to June 2005 in Texas, studied whether the inclusion of homicide survivors (i.e., victims' relatives and friends) at executions beginning in January 1996 (Texas Department of Criminal Justice, 2017b) had influenced death row inmates' last words. The authors found that prisoners were more likely to make a final statement from January 1996 onwards (82% of the prisoners made a final statement from this date onwards; prior to this date, 68% made a final statement). Further, Rice et al. (2009) showed that death row inmates' last words were more likely to contain expressions of guilt and repentance after victim witnesses were provided the opportunity to attend executions in January 1996.

Taken together, the reviewed text analysis studies of executed death row inmates' last words demonstrated that they contained surprisingly more positive than negative emotional expressions, with final statements' content themes revolving most strongly around expressions of love and appreciation, messages to relevant social others, and religious beliefs (see also Ward, 2010; Johnson et al., 2014, for general overviews). That is, prisoners' final statements spoken moments prior to death seemed to focus on aspects, including social connections and religion, that make life (and death) meaningful (see e.g., Smith, 2017).

### Affective experiences and meaning-making in executed death row inmates' last words compared with those of people's conceptions of death and dying

Do the affective experiences and meaning-making attempts in the last words of executed prisoners differ from those expressed by people imagining death and dying? So far, research evidence comparing death row inmates' actual last words spoken in the face of imminent death with people's ideas about how they would feel when asked to imagine their own impending death is extremely scarce. In a recent study, Goranson et al. (2017) compared affective expressions in executed Texas death row inmates' last words with those from people's imaginations of imminent death by execution. Using both LIWC and affect ratings by human coders, the authors showed that executed prisoners' last words contained significantly more expressions of positive affect and fewer expressions of negative affect than people's simulated written last statements when asked to imagine what it would be like to face execution the next day. Further, the results indicated that compared with people's simulated last statements, death row inmates' last words contained significantly higher rates of words from the LIWC categories social connection and religion. In addition, the differences in positive and negative affect between death row inmates' last words and people's simulated last statements could be partially explained by death row inmates' increased use of social-connection and religion words (Goranson et al., 2017). Similarly, comparing the last words of executed prisoners with people's general ideas about death and dying, we found that death row inmates' final statements contained a significantly larger proportion of words reflecting positive affect than the writings of people contemplating their own death (cf. Hirschmüller and Egloff, 2016, Table 3; Kashdan et al., 2014).

Other research has explored the conceptions of death and dying of people in their end-of-life phase with the help of interviews. For example, terminally ill hospice patients, when interviewed about their thoughts and feelings about their forthcoming death, explained that they felt sorrowful about leaving life, but the majority of them expressed that they were not anxious about their death. They further stated that one way of coping with their death included focusing on the positive aspects of their lives (Moestrup and Hansen, 2015). Further, Arnold (2011) found that the most prevalent themes in hospice patients' interviews included references to other people and time-related comments expressing and locating present experiences in their life course. Similarly, in interviews with patients with life-threatening illnesses facing imminent death, these patients described the strength they drew from social relationships and expressed needs for love, meaning, purpose, and often spirituality (Murray et al., 2004). In interviews with old people diagnosed with cancer, Thomé (2003) found that they seemingly accepted death and the finiteness of life but feared dying and related aspects (e.g., suffering, the health care system failing to meet their needs; for a general overview of old people's views of their forthcoming death and dying, see Hallberg, 2004). Altogether, the findings indicate that terminally ill people seem to fear the process of dying more than death itself.

Moreover, a large body of research has used mortality salience manipulations to study how people imagine death and dying in the laboratory (for overviews, see e.g., Burke et al., 2010; Lambert et al., 2014; Pyszczynski et al., 2015). Most of these studies have used death essay questions—asking participants to write about the emotions that thoughts of their own death arouse in them and what they think will happen to them as they physically die—and compared participants' answers with people's writings about non-death-related control topics (Burke et al., 2010). For example, Kashdan et al. (2014) found that the writings of people who had answered questions about their own death included more positive emotion words than control groups' writings about dental pain, uncertainty, or meaninglessness. Further studies have emphasized the role of effects of mortality salience on relational strivings and the relevance of close relationships (Mikulincer et al., 2003) and religious beliefs (Vail et al., 2010; Halberstadt and Jong, 2014). Despite the advantages of experimental control and the opportunity to make comparisons with control groups, mortality salience manipulations in which individuals contemplate their own deaths, however, most likely differ in the degree to which they provoke the feelings that being confronted with one's actual death does.

In sum, both death row inmates' last words in the face of death and people's conceptions of death and dying, despite the incomparability of the situations and different methods and samples, reveal a considerable amount of emotional positivity and the importance of social connections and spirituality. These aspects appeared to be even more pronounced in executed prisoners' final statements compared with people who were asked to imagine they were facing death by execution or death in general (Hirschmüller and Egloff, 2016; Goranson et al., 2017).

## Beyond death row inmates' last words: situating the reviewed findings in the research context of the study of language use preceding death

Each of the reviewed text analysis studies in this article of last words spoken by death row inmates executed since 1982 in Texas has valuably contributed to the understanding of people's finite accounts in the face of death and dying. Despite a focus on different research questions and methods, through the use of computerized text analysis and/or human coders to identify a varying number of content themes—as such not easily comparable—each finding has added to the comprehensive picture of death row inmates' final utterances, revealing what people chose to express to others in the final moments of their lives. The majority of the studies reviewed in this article have focused exclusively on the text of last statements from different time periods but excluded other potential factors from their analyses, such as demographic information for the specific sample (Cunningham and Vigen, 2002) or information about the nature of the crime presented for each prisoner on the Texas Department of Criminal Justice (2017a) website. Moreover, as the findings indicate, inmates' last words seem to be shaped at least in part by the extent to which the executions were public and occurred in the presence of witnesses (see Rice et al., 2009). However, the individual influence of the people who were actually present at the execution (i.e., inmates' and/or victims' relatives and friends) on the delivery of last words remains unclear, as no comprehensive record is available for the specific circumstances of individual executions. In addition, it is noteworthy that about 20% of the inmates executed within the time period from 1982 to 2015 had declined to make a final statement. As the explicit reasons are not provided, it remains unclear why these inmates chose to forgo their last opportunity to speak final words.

**Figure 2:**
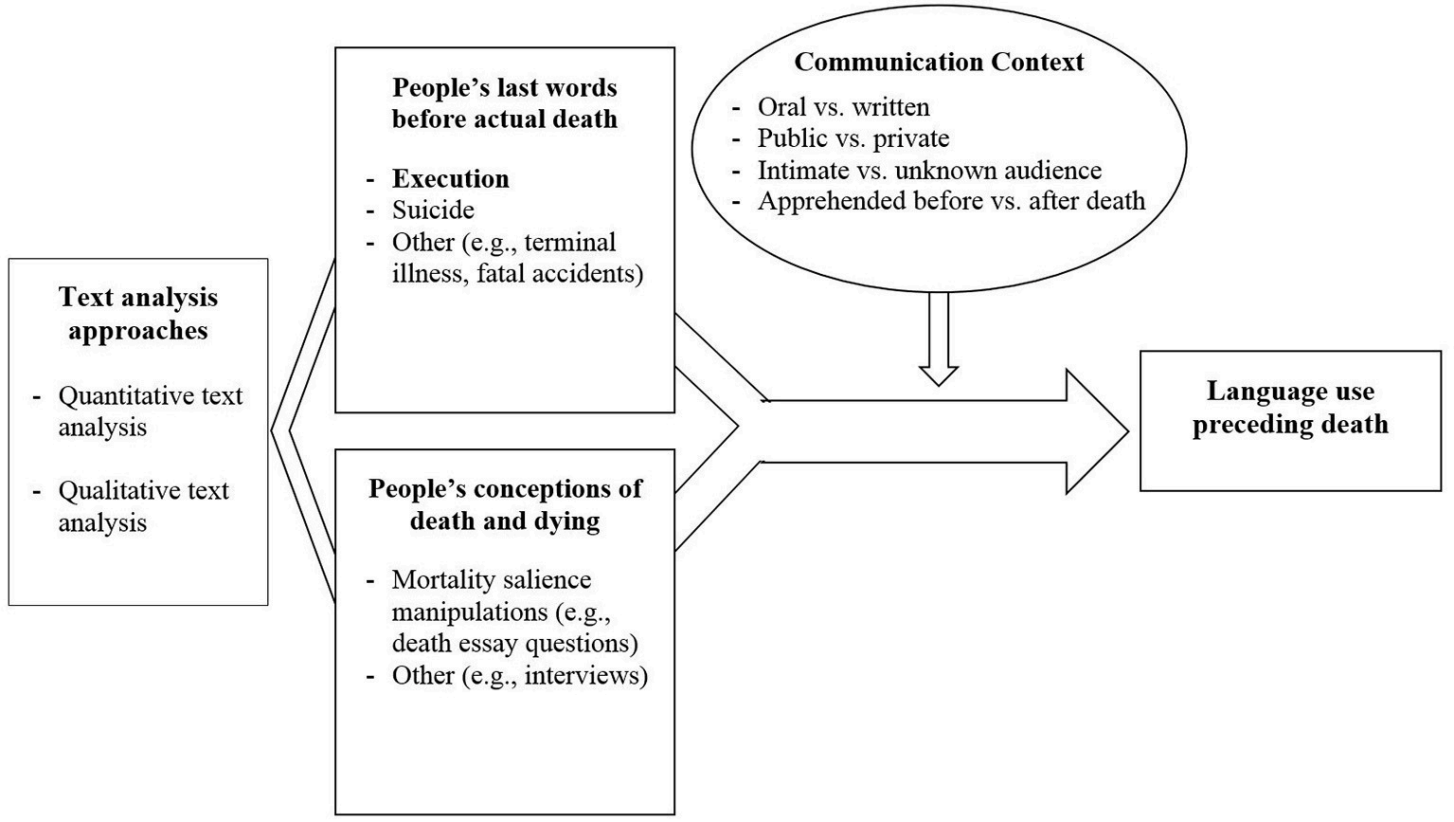
Situating text analysis studies of death row inmates' last words into a framework of methodological approaches for the study of language use preceding death.

What makes death row inmates' last words unique is that there is no doubt that these words are an individual's final message to the outside world. Death row inmates usually spend a number of years on death row prior to their execution. As such, their last words are spoken after lengthy periods of time in which the prisoners were able to consider and prepare for the definite end of their lives, and this process may have brought some sense of peace or acceptance of the inevitability of death (cf. Kübler-Ross, 1969). Moreover, as humans, we generally remain uncertain throughout our lives about when and how death will occur. Death row inmates, however, are in a situation in which they are aware of the date, time, place, and manner in which they will die. In essence, this distinguishes inmates on death row from people suffering from a terminal illness, older people, or people contemplating death in a variety of experimental settings and in general (see Figure 2). Thus, the reviewed findings cannot easily be generalized to people facing greater uncertainty about death and dying.

Research on other forms of communication from people who are dying has broadened the understanding of the feelings and themes individuals choose to express to others under various circumstances of death and dying (e.g., quick vs. lingering trajectories; cf. Kastenbaum, 2016, see Figure 2). Text analysis studies of suicide notes, for example, have revealed a predominance of positive over negative emotional expressions (Tuckman et al., 1959) and have found that, along with many instructions and a great deal of information for others, the most frequently expressed emotions include love, thankfulness, hopelessness, and guilt (e.g., Pestian et al., 2012; Desmet and Hoste, 2013). Written suicides notes (left behind by 10–43% of suicides; Pestian et al., 2012) differ from the last words publicly spoken before execution in that they are privately composed and meant to be apprehended by the bereaved after death. Providing psychological insights into the last hours before death, Gunn and Lester (2012) found that Twitter postings of a young girl in the 24 hours before her suicide increased in positive emotion word use and changed from focusing on the self to others. Similarly, terminally ill individuals' blog posts were found to contain a surprisingly large amount of emotional positivity and a significant increase in positive affect as patients approached death (Goranson et al., 2017). These final verbal communications—though differing in the communication context from executed prisoners' spoken last words (e.g., oral vs. written; public vs. private; cf. Balon and Rimé, 2016), contained a large degree of positive affect, similar to inmates' final statements, which seemed to increase in positivity as they approached death.

Taken together, the intriguing phenomenological exploration of people's last words in the unique context of dying by execution provides a valuable link to understanding people's communication to others in the face of imminent death. Despite the existing moral, legal, and ethical issues surrounding capital punishment (Bohm, 2016) and researchers' constraints due to their reliance on publicly available information, the continuing examination of these prisoners' final statements can contribute to a better understanding of human mortality in general. In the following sections, we will outline unanswered research questions and describe how these prisoners' last statements may be more comprehensively analyzed in future studies.

## Broadening the scope: open questions and future directions

The thoughts and emotions expressed by death row inmates in the acute powerless situation of dying by execution may be considered in the broad context of **emotion regulation** (Gross, 2015) and the related field of coping (Lazarus and Folkman, 1984). Emotion regulation often serves the intrinsic function of regulating one's own emotions, but it can also be interpersonal in nature as it can be aimed at regulating another person's emotions (Rimé, 2007; Zaki and Williams, 2013). One particularly interesting and understudied aspect of death row inmates' last words pertains to the intra- versus interpersonal nature and function of emotion expression and regulation. Beyond the issues of how and what death row inmates linguistically express as summarized in this review, an interesting open question for future research concerns the extent to which the expressed contents are both intended to regulate and effective at regulating inmates' own and/or the addressees' emotions, respectively. For example, using qualitative text analysis, human coders could rate the extent to which last words appear to be spoken to regulate death row inmates' own emotions as well as the emotions of their execution witnesses (cf. Schuck and Ward, 2008). Moreover, as language studies of trauma narratives have suggested that the use of death words can indicate a person's perceived loss of all autonomy during traumatic events (see Alvarez-Conrad et al., 2001), death and dying words in inmates' final statements could be more comprehensively analyzed to gain a better understanding of inmates' psychological state and effectiveness in regulating their own emotions when facing execution.

KEY CONCEPT 4 **Emotion regulation**

Automatic or controlled processes by which people influence which emotions they have, when they have these emotions, and how they experience and express them.

Moreover, future studies may place a stronger focus on the interpersonal nature and function of inmates' last words. Exploring execution witnesses' accounts to a greater extent could broaden the understanding of whether witnessing last statements can help homicide victims' relatives and friends cope with the crime and find closure (see e.g., Gross and Matheson, 2003; Vollum and Longmire, 2007). An important question that could be more comprehensively studied in future research is how inmates' apologies and expressions of remorse, guilt, and moral sentiment are related to forgiveness and psychological adjustment in victims' families and friends. To this end, for example, moral language use in prisoners' last statements (e.g., Sagi and Dehghani, 2014) and indicators of well-being and mental health in homicide survivors' accounts (e.g., Schwartz et al., 2016; Guntuku et al., 2017) may be assessed using recent automated quantitative text analysis approaches. Further, another important and interesting aspect for future research concerns the effect of death row inmates' last words on the general public's social perceptions and judgments of executed prisoners. For instance, by conducting psychological laboratory studies and by surveying the US population in the real world, researchers can more comprehensively study how perceiving inmates' last words and media accounts of prisoners' executions (cf. Miller and Hunt, 2008) shape people's social judgments of executed death row inmates and public opinions about the death penalty.

In addition, future research integrating human annotations and automated text analysis approaches (cf. Schaefer Ziemer and Korkmaz, 2016) could more comprehensively aim to identify the specific positive and negative emotions expressed in inmates' final statements. However, it is important to keep in mind that the final words publicly expressed by death row inmates represent a linguistic and content-related snapshot that reflects only one channel of emotion expression and regulation (Reilly and Seibert, 2003). As such, the extent and types of linguistically expressed emotions may overlap only to a certain degree with the emotions inmates experience and other emotion response tendencies including physiological responses, vocal and facial expressions, and body language (Mauss and Robinson, 2009). An important aspect that remains unresolved pertains to the question of whether death row inmates actually experience more positive than negative emotions in the final moments before death. Do death row inmates' emotion regulation goals in their final moments of life mainly involve decreasing negative or increasing positive affect? In addition, to what extent do they use other behavioral means to regulate emotion such as singing, crying, or silently praying?

Another important open question is whether and to what extent the emotions that are experienced and expressed in the final moments of life are influenced by the beginning and time course of emotion regulation (cf. Sheppes and Gross, 2011) and death row inmates' previous efforts to cope with the inevitability of impending death during their time on death row. A systematic longitudinal exploration of death row inmates' thoughts and feelings about life and death on death row along with their final statements might offer a more comprehensive perspective on their psychological coping with impending death and dying. Future studies could analyze other forms of communication composed by death row inmates longer before their death (e.g., inmates' poetry) to examine whether inmates' affect increased in positivity as they moved closer to death as suggested by recent research (cf. Goranson et al., 2017). In addition, valuable personal accounts including inmates' letters (Arriens, 2004), interviews (Dicks, 2013), and autobiographical writings (Abu-Jamal, 1996; Rossi, 2004), giving voice to particular inmates and their specific situation of life on death row, can broaden our understanding of how death row inmates cope with death and dying.

## Practical implications

In the US, the only Western culture to still enforce executions, death is a topic that is greatly avoided (Kastenbaum, 2016). The fact that there are still executions taking place today calls for a better understanding of death row inmates' unique situation, and understanding these aspects provides a knowledge base that can inform correctional staff and death row inmates' families and friends how to approach these people about death and dying and provide them with high-quality support and guidance. It is important to mention that the consequences of the death penalty and executions are not limited to the prisoners and the loved ones, homicide survivors, and correctional staff they leave behind; rather, the list also includes members of the media, the legal community, and all of us as a society. The last words of executed death row inmates can be seen as an indication of what is important for people to communicate to others before imminent death. As such, these final utterances can inform psychologists, health care professionals, and others who deal with death and dying in professional contexts, but also all of us in general, about dying people's communication needs. As death is certain for us all, there is a continuing interest and need to review and refine our conceptions of death and dying and our end-of-life communication needs and themes. Avoiding talking about death probably makes us even more anxious about it, and a more open conversation about death and a better understanding of life's eventual end may remind us to enjoy and live life more meaningfully (Ma-Kellams and Blascovich, 2012; Vail et al., 2012).

## Conclusions

Inmates' final words spoken moments before their executions—a scheduled final act of verbal communication that is publicly witnessed—provide an indication of individuals' affective states and meaning-making attempts in the face of imminent actual death. With the use of automated and human coded text analysis, research has revealed a surprisingly strong predominance of positive emotional sentiments in death row inmates' last words, accompanied by expressions of love, affection, apologies, and religion, reflecting death row inmates' existential communication concerns in their final minutes of life. Although prisoners' final statements appeared to be less negative than expected by people imagining their own impending death, the findings should not be interpreted to mean that dying people view death as a wholly positive experience: Being less negative is not the same as wanting or welcoming death. Our fear of death is as inevitable as the event itself, and these people who died by execution feared death. Their last words simply did not reflect as much fear as people imagining death would expect. Despite the fact that this intriguing phenomenological exploration of these last statements has informed (even our own) psychological research, we agree with the many opponents of capital punishment (Yorke, 2016) that the death penalty practice is inhumane and totally wrong for many different reasons. One of these reasons was best expressed by a chaplain who has witnessed 95 of the executions in Texas himself: “How can Texas kill people to teach other people that killing people is wrong?” (Pickett, 2013).

## Author contributions

SH drafted the manuscript under the guidance of BE. Both authors approved the final version of the manuscript for submission and publication.

### Conflict of interest statement

The authors declare that the research was conducted in the absence of any commercial or financial relationships that could be construed as a potential conflict of interest.

## References

[B1] Abu-Jamal M. (1996). Live From Death Row. New York, NY: Harper Perennial.

[B2] Alvarez-Conrad J. Zoellner L. A. Foa E. B. (2001). Linguistic predictors of trauma pathology and physical health. Appl. Cogn. Psychol. 15, 159–170. 10.1002/acp.839

[B3] Arnold B. L. (2011). Mapping hospice patients' perception and verbal communication of end-of-life needs: an exploratory mixed methods inquiry. BMC Palliat. Care 10:1. 10.1186/1472-684X-10-1. 21272318 PMC3038142

[B4] Arriens J. (2004). Welcome to Hell: Letters and Writings from Death Row. Boston, MA: Northeastern University Press.

[B5] Balon S. Rimé B. (2016). Lexical profile of emotional disclosure in socially shared versus written narratives. J. Lang. Soc. Psychol. 35, 345–373. 10.1177/0261927X15603425

[B6] Banner S. (2003). The Death Penalty: An American History. Cambride, MA: Havard University Press.

[B7] Bantum E. O. Owen J. E. (2009). Evaluating the validity of computerized content analysis programs for identification of emotional expression in cancer narratives. Psychol. Assess. 21, 79–88. 10.1037/a0014643 19290768

[B8] Becker E. (1973). The Denial of Death. New York, NY: Free Press.

[B9] Bedeau H. Cassell P. (2004). Debating the Death Penalty: Should America have Capital Punishment? New York, NY: Oxford University Press.

[B10] Bohm R. M. (2016). Deathquest: An Introduction to the Theory and Practice of Capital Punishment in the United States. New York, NY: Routledge.

[B11] Burke B. L. Martens A. Faucher E. H. (2010). Two decades of terror management theory: a meta-analysis of mortality salience research. Person. Soc. Psychol. Rev. 14, 155–195. 10.1177/1088868309352321 20097885

[B12] Cooney M. Phillips S. (2013). With god on one's side: the social geometry of death row apologies. Sociol. Forum 28, 159–178. 10.1111/socf.12007

[B13] Cunningham M. D. Vigen M. P. (2002). Death row inmate characteristics, adjustment, and confinement: a critical review of the literature. Behav. Sci. Law 20, 191–210. 10.1002/bsl.473 11979498

[B14] Death Penalty Information Center (2017). Facts About The Death Penalty. Available online at: https://deathpenaltyinfo.org/documents/FactSheet.pdf (Accessed November 15, 2017).

[B15] Desmet B. Hoste V. (2013). Emotion detection in suicide notes. Expert Syst. Appl. 40, 6351–6358. 10.1016/j.eswa.2013.05.050

[B16] Dicks S. (2013). Death Row: Interviews with Inmates, Their Families and Opponents of Capital Punishment. Jefferson, NC: McFarland & Co Inc.

[B17] Eaton J. (2014). Honor on death row: apology, remorse, and the culture of honor in the U.S. South. SAGE Open 4, 1–9. 10.1177/2158244014529777

[B18] Eaton J. Theuer A. (2009). Apology and remorse in the last statements of death row prisoners. Justice Q. 26, 327–347. 10.1080/07418820802245078

[B19] Fellows I. (2015). Package ‘Wordcloud’ (Version 2.5). Available online at: https://cran.r-project.org/web/packages/wordcloud/wordcloud.pdf (Accessed November 20, 2017).

[B20] Foley S. R. Kelly B. D. (2007). The psychological concomitants of capital punishment: thematic analysis of last statements from death row. Am. J. Forensic Psychiatry 28, 7–13.

[B21] Goranson A. Ritter R. S. Waytz A. Norton M. I. Gray K. (2017). Dying is unexpectedly positive. Psycholo. Sci. 28, 988–999. 10.1177/0956797617701186 28569605

[B22] Gross J. J. (2015). Emotion regulation: current status and future prospects. Psychol. Inquiry 26, 1–26. 10.1080/1047840X.2014.940781

[B23] Gross S. K. Matheson D. J. (2003). What they say at the end: capital victims' families and the press. Cornell Law Rev. 88, 486–516. 10.2139/ssrn.415081

[B24] Gunn J. F. Lester D. (2012). Twitter postings and suicide: an analysis of the postings of a fatal suicide in the 24 hours prior to death. Suicidologi 17, 28–30. Available online at: https://www.journals.uio.no/index.php/suicidologi/article/view/2173/2036

[B25] Guntuku S. C. Yaden D. B. Kern M. L. Ungar L. H. Eichstaedt J. C. (2017). Detecting depression and mental illness on social media: an integrative review. Curr. Opin. Behav. Sci. 18, 43–49. 10.1016/j.cobeha.2017.07.005

[B26] Halberstadt J. Jong J. (2014). Scaring the bejesus into people: the role of religious belief in managing implicit and explicit anxiety, in Motivation and its Regulation: The Control Within, eds Forgas J. P. Harmon-Jones E. (New York, NY: Psychology Press), 331–350.

[B27] Hallberg I. R. (2004). Death and dying from old people's point of view. A literature review. Aging Clin. Exp. Res. 16, 87–103. 10.1007/BF03324537 15195983

[B28] Heflick N. A. (2005). Sentenced to die: last statements and dying on death row. Omega-Journal of Death and Dying 51, 323–336. 10.2190/96X8-FLUT-TCLH-EL71

[B29] Hirschmüller S. Egloff B. (2016). Positive emotional language in the final words spoken directly before execution. Front. Psychol. 6:1985. 10.3389/fpsyg.2015.01985 26793135 PMC4710806

[B30] Hood R. Hoyle C. (2015). The Death Penalty: A Worldwide Perspective. New York, NY: Oxford University Press.

[B31] Howell T. B. (1809). Cobbett's Complete Collection of State Trials and Proceedings for High Treason and Other Crimes and Misdemeanors from the Earliest Period to the Present Time. London: R. Bagshaw.

[B32] Johnson R. Kanewske L. C. Barak M. (2014). Death row confinement and the meaning of last words. Laws 3, 141–152. 10.3390/laws3010141

[B33] Kahn J. H. Tobin R. M. Massey A. E. Anderson J. A. (2007). Measuring emotional expression with the linguistic inquiry and word count. Am. J. Psychol. 120, 263–286. 10.2307/20445398 17650921

[B34] Kashdan T. B. DeWall C. N. Schurtz D. R. Deckman T. Lykins E. L. B. Evans D. R. (2014). More than words: contemplating death enhances positive emotional word use. Person. Ind. Diff. 71, 171–175. 10.1016/j.paid.2014.07.035

[B35] Kastenbaum R. (2000). The Psychology of Death. New York, NY: Springer.

[B36] Kastenbaum R. J. (2016). Death, Society, and Human Experience. New York, NY: Routledge.

[B37] Kelly B. D. Foley S. R. (2013). Love, spirituality, and regret: Thematic analysis of last statements from death row, Texas (2006–2011). J. Am. Acad. Psychiatry Law 41, 540–550. Available online at: https://pdfs.semanticscholar.org/8701/6e5577cd8ac4ad85339cfb4d8034adc71a9c.pdf 24335328

[B38] Kübler-Ross E. (1969). On Death and Dying. New York, NY: Macmillan.

[B39] Kuckartz U. (2014). Qualitative Text Analysis: A Guide to Methods, Practice and Using Software. Thousand Oaks, CA: Sage.

[B40] Lambert A. J. Eadeh F. R. Peak S. A. Scherer L. D. Schott J. P. Slochower J. M. (2014). Toward a greater understanding of the emotional dynamics of the mortality salience manipulation: revisiting the “affect-free” claim of terror management research. J. Person. Soc. Psychol. 106, 655–678. 10.1037/a0036353 24749817

[B41] Lazarus R. S. Folkman S. (1984). Stress, Appraisal, and Coping. New York, NY: Springer.

[B42] Ma-Kellams C. Blascovich J. (2012). Enjoying life in the face of death: East-west differences in responses to mortality salience. J. Person. Soc. Psychol. 103, 773–786. 10.1037/a0029366 22823291

[B43] Marvin F. R. (1901). The Last Words (Real and Traditional) of Distinguished Men and Women Collected from Various Sources. New York, NY: Fleming H. Revell.

[B44] Mauss I. Robinson M. (2009). Measures of emotion: a review. Cogn. Emot. 23, 209–237. 10.1080/02699930802204677 19809584 PMC2756702

[B45] Mehl M. R. (2006). Quantitative text analysis, in Handbook of Multimethod Measurement in Psychology, eds Eid M. Diener E. (Washington, DC: American Psychological Association), 141–156.

[B46] Mikulincer M. Florian V. Hirschberger G. (2003). The existential function of close relationships: introducing death into the science of love. Person. Soc. Psychol. Rev. 7, 20–40. 10.1207/S15327957PSPR0701_2 12584055

[B47] Miller K. S. Hunt S. A. (2008). Exit stage left: a dramaturgical analysis of media accounts of executions in America. J. Crim. Justice Popular Cult. 15, 189–217. Available online at: https://www.albany.edu/scj/jcjpc/vol15is2/MillerHunt.pdf

[B48] Moestrup L. Hansen H. P. (2015). Existential concerns about death: a qualitative study of dying patients in a danish hospice. Am. J. Hospice Palliative Med. 32, 427–436. 10.1177/1049909114523828 24595321

[B49] Murray S. A. Kendall M. Boyd K. Worth A. Benton T. F. (2004). Exploring the spiritual needs of people dying of lung cancer or heart failure: a prospective qualitative interview study of patients and their carers. Palliat. Med. 18, 39–45. 10.1191/0269216304pm837oa 14982206

[B50] Neimeyer R. A. (2015). Death Anxiety Handbook: Research, Instrumentation, and Application. New York, NY: Routledge.

[B51] Pennebaker J. W. Booth R. J. Boyd R. L. Francis M. E. (2015). Linguistic Inquiry and Word Count: LIWC 2015. Austin, TX: Pennebaker Conglomerates Available online at: www.LIWC.net.

[B52] Pennebaker J. W. Booth R. J. Francis M. E. (2007a). Linguistic Inquiry and Word Count: LIWC 2007. Austin, TX: LIWC.

[B53] Pennebaker J. W. Chung C. K. Ireland M. Gonzales A. Booth R. J. (2007b). The Development and Psychometric Properties of LIWC2007. Austin, TX: LIWC.net.

[B54] Pestian J. P. Matykiewicz P. Linn-Gust M. (2012). What's in a note: construction of a suicide note corpus. Biomed. Inform. Insights 5, 1–6. 10.4137/BII.S10213 23170067 PMC3500150

[B55] Pickett C. (2013). Texas prison chaplain: 'I've come to see the death penalty as totally wrong. Available online at: https://www.theguardian.com/world/2013/jun/27/capital-punishment-texas-pickett (Accessed November 20, 2017).

[B56] Pyszczynski T. Solomon S. Greenberg J. (2015). Thirty years of terror management theory: from genesis to revelation, in Advances in Experimental Social Psychology, Vol. 52 eds Olson J. M. Zanna M. P. (Waltham, MA: Academic Press), 1–70.

[B57] Reilly J. Seibert L. (2003). Language and emotion, in Handbook of Affective Sciences, eds Davidson R. J. Sherer K. R. Goldsmith H. H. (New York, NY: Oxford University Press), 535–558.

[B58] Rice S. K. Dirks D. Exline J. J. (2009). Of guilt, defiance, and repentance: evidence from the Texas death chamber. Justice Q. 26, 295–326. 10.1080/07418820802178063

[B59] Rimé B. (2007). Interpersonal emotion regulation, in Handbook of Emotion Regulation, ed Gross J. J. (New York, NY: Guilford), 466–485.

[B60] Rossi R. M. (2004). Waiting to Die: Life on Death Row. New York, NY: Vision Paperbacks.

[B61] Sagi E. Dehghani M. (2014). Measuring moral rhetoric in text. Soc. Sci. Comput. Rev. 32, 132–144. 10.1177/0894439313506837

[B62] Schaefer Ziemer K. Korkmaz G. (2016). Human vs. automated text analysis: Estimating positive and negative affec, in HT'16 Proceedings of the 27th ACM Conference on Hypertext & Social Media. New York, NY: ACM, 309–314.

[B63] Schuck A. R. T. Ward J. (2008). Dealing with the inevitable: strategies of self-presentation and meaning construction in the final statements of inmates on Texas death row. Discourse Soc. 19, 43–62. 10.1177/0957926507083687

[B64] Schwartz H. A. Sap M. Kern M. L. Eichstaedt J. C. Kapelner A. Agrawal M.. (2016). Predicting individual well-being through the language of social media, in Biocomputing 2016: Proceedings of the Pacific Symposium (Kohala Coast), 516–527. 26776214

[B65] Sheppes G. Gross J. J. (2011). Is timing everything? Temporal considerations in emotion regulation. Person. Soc. Psychol. Rev. 15, 319–331. 10.1177/1088868310395778 21233326

[B66] Smith E. E. (2017). The Power of Meaning: Crafting a Life that Matters. New York, NY: Crown.

[B67] Texas Department of Criminal Justice (2012). Execution Procedure. Available online at: https://static.texastribune.org/media/documents/TDCJ_Execution_Protocol_07-09-2012_Final.pdf (Accessed October 30, 2017).

[B68] Texas Department of Criminal Justice (2017a). Death Row Information. Available online at: http://www.tdcj.state.tx.us/death_row/dr_executed_offenders.html (Accessed October 30, 2017).

[B69] Texas Department of Criminal Justice (2017b). Victim Services Division–Viewing Executions. Available online at: http://www.tdcj.state.tx.us/divisions/vs/viewing_executions.html (Accessed November 10, 2017).

[B70] Thomé B. (2003). Living with Cancer in Old Age: Quality of Life and Meaning, Doctoral thesis, Lund University, Lund.

[B71] Tuckman J. Kleiner R. J. Lavell M. (1959). Emotional content of suicide notes. Am. J. Psychiatry 116, 59–63. 10.1176/ajp.116.1.59 13661450

[B72] Vail K. E. Juhl J. Arndt J. Vess M. Routledge C. Rutjens B. T. (2012). When death is good for life: considering the positive trajectories of terror management. Person. Soc. Psychol. Rev. 16, 303–329. 10.1177/1088868312440046 22490977

[B73] Vail K. E. Rothschild Z. K. Weise D. R. Solomon S. Pyszczynski T. Greenberg J. (2010). A terror management analysis of the psychological functions of religion. Person. Soc. Psychol. Rev. 14, 84–94. 10.1177/1088868309351165 19940284

[B74] Vollum S. (2008). Last Words and the Death Penalty: Voices of the Condemned and Their Co-Victims. El Paso, TX: LFB Scholarly.

[B75] Vollum S. Longmire D. R. (2009). Giving voice to the dead: last statements of the condemned. Contemp. Justice Rev. 12, 5–26. 10.1080/10282580802681576

[B76] Vollum S. Longmire D. R. (2007). Covictims of capital murder: statements of victims' family members and friends made at the time of execution. Viol. Vict. 22, 601–619. 10.1891/088667007782312131 18064972

[B77] Ward J. (2010). Communication from the condemned. Psychologist 23, 724–727. Available online at: https://thepsychologist.bps.org.uk/getfile/1608

[B78] Yorke J. (2016). Against the Death Penalty: International Initiatives and Implications. New York, NY: Routledge.

[B79] Zaki J. Williams W. C. (2013). Interpersonal emotion regulation. Emotion 13, 803–810. 10.1037/a0033839 24098929

